# Adrenarche and pubarche in girls with turner syndrome during growth-promoting therapy with human growth hormone

**DOI:** 10.1186/s12902-019-0333-z

**Published:** 2019-01-18

**Authors:** Helmuth G. Dörr, Theresa Penger, Michaela Marx, Manfred Rauh, Patricia G. Oppelt, Thomas K. M. Völkl

**Affiliations:** 1 Paediatric Endocrinology, University Hospital of Erlangen, Loschgestr. 15, 91054 Erlangen, Germany; 2 Laboratory Medicine, University Hospital of Erlangen, Loschgestr. 15, 91054 Erlangen, Germany; 3 Department of Paediatrics, and Paediatric Gynaecology, University Hospital of Erlangen, Loschgestr. 15, 91054 Erlangen, Germany; 4 Department of Gynaecology and Obstetrics, University Hospital of Erlangen, Loschgestr. 15, 91054 Erlangen, Germany

**Keywords:** Turner syndrome, DHEAS, Adrenarche, Primary ovarian insufficiency

## Abstract

**Background:**

Data on adrenarche and pubarche in girls with Turner syndrome (TS) are inconsistent in the literature.

**Methods:**

The cohort consisted of 94 girls and young women with TS born between 1971 and 2001 (age range: 3.1–23.2 yrs.), who were treated with human growth hormone and regularly presented at our outpatient clinic every 4 to 6 months.The longitudinal data of all patients were ascertained retrospectively from patient charts. The data collection ended in January 2016. Adrenarche was assessed by serum DHEAS levels and pubertal status by Tanner stages. Pubarche was defined as the appearance of pubic hair (PH2), whereas spontaneous puberty was defined as Tanner stage B2. The patients were retrospectively subdivided in two groups with regard to pubertal development: group 1 (*n* = 21) with spontaneous puberty and group 2 (*n* = 70) with induced puberty. Since blood samples were not taken at every visit, we generated seven groups according to the age of the children at which the blood samples were taken: 3–5, 5–7, 7–9, 9–11, 11–13, 13–15, and 15–17 yrs. Serum DHEAS and follicle-stimulating hormone (FSH) levels were measured by chemiluminescence immunoassay and compared with those of a control group of healthy girls.

**Results:**

Adrenarche started in TS girls between 5 and 7 years. TS girls had higher DHEAS levels than the control group, with statistically significant differences in the age groups 7 to 17 years. No differences were determined between the TS girls with spontaneous puberty and those with POI. TS girls in group 2 reached the Tanner stages PH2 (*p* < 0.04), PH3 (*p* < 0.01), PH4 and PH5 (*p* < 0.001) markedly later than TS girls in group 1.

**Conclusions:**

The onset of adrenarche in girls with TS undergoing GH therapy does not differ from that in healthy girls. However, adrenarche is more pronounced in girls with TS. There is no difference in DHEAS levels between the TS girls with spontaneous puberty and the TS girls with primary ovarian insufficiency (POI), while the tempo of pubarche is markedly slower in the girls with POI.

## Background

Turner syndrome (TS) affects approximately 1 in 2500 live-born females. It is characterized by loss or structural anomalies of an X chromosome. Multiple organ systems are affected but the main clinical features are short stature and gonadal dysgenesis associated with gonadal failure [1–3]. Data on spontaneous puberty with normal menarche are inconsistent in the literature and varied from 10 to 31% [4, 5].

Adrenarche defines the start of the physiological secretion of adrenal androgens, whereas the term pubarche describes the phenotypic result of adrenarche. The best markers of adrenarche are the increasing serum concentrations of dehydroepiandrosterone (DHEA) and its sulfate conjugate DHEAS, typically at 5 to 6 years of age in healthy girls. Many factors have been identified in the regulation of adrenarche in the past, such as normal ACTH secretion, increasing adrenal 17,20-lyase activity and serum IGF-1 (Insulin-like Growth Factor 1) concentrations [6–8]. Changes in insulin sensitivity and adiposity might also be involved in adrenal androgen synthesis [9]. It has also been speculated that treatment with growth hormone (GH) might affect adrenarche. A case report suggested that GH excess may result in premature adrenarche [10], whereas no changes in DHEAS levels were found in children with GH deficiency after 6 months of GH therapy [11], or in boys with non-GH deficient short stature [12]. However, there are still many unknown factors in the regulation of adrenarche.

Gonadarche and adrenarche are regarded as processes that are independent of each other [13–15]. Therefore, adrenarche should not be affected in girls with TS and primary ovarian insufficiency (POI). Data on adrenarche in the literature are scarce and inconsistent. Markedly elevated DHEAS concentrations suggest an exaggerated adrenarche in TS girls [16]. Both normal adrenarche [15, 17] and early adrenarche with delayed pubarche [18] were found in TS girls with POI. No details regarding GH treatment of the girls with TS are found in the paper by Martin et al. [18].

The aim of the present study was to examine the course of adrenarche and pubarche in girls with TS during therapy with GH, both those with spontaneous onset of puberty and those without spontaneous puberty due to ovarian insufficiency.

## Methods

The whole cohort consisted of 94 girls and young women with TS born between 1971 and 2001. 52% of the girls had karyotype 45 X. All patients were treated with human growth hormone (GH) and regularly presented at our outpatient clinic every 4 to 6 months. GH therapy was started at age 8.2 ± 3.3 (mean ± SD) years (age range: 3.1–21.3 yrs.) and ended at age 14.6 ± 1.61 years (age range: 9.4–23.2 yrs.). The mean duration of therapy was 6.0 ± 2.7 years. The mean initial GH dose was 0.31 ± 0.04 mg/kg body weight and week (range: 0.14–0.44 mg/kg body weight and week).

The longitudinal data of all patients were ascertained retrospectively from patient charts. Data collection ended in January 2016. The aim of the study was to analyze the course of adrenarche and pubarche in girls with TS during therapy with growth hormone. Therefore, the patients were divided retrospectively into two groups regarding pubertal development: group 1 (*n* = 21) with spontaneous onset of puberty and group 2 (*n* = 70) with induced puberty due to primary ovarian insufficiency (POI). Assessment of pubertal stage was not possible in three girls at the time of analysis due to their their young age. Since blood samples were not taken at every visit, we generated seven groups according to the age of the children at which the blood samples were taken: 3–5, 5–7, 7–9, 9–11, 11–13, 13–15, and 15–17 yrs. BMI assessment was based on German data [19]. Pubertal maturation stage was documented according to Marshall and Tanner [20]. Pubarche was defined as the appearance of pubic hair (PH2); spontaneous puberty was defined as breast development Tanner stage B2. TS girls with POI had no spontaneous breast development and markedly elevated serum FSH levels. The BMI of the girls with TS lay between the 10th and 90th percentile during GH therapy: hence, it was possible to use the data of the German nationwide representative longitudinal and cross-sectional study on the health of children and adolescents (KIGGS Report) as the reference cohort for age assessment at the different stages of pubic hair in the TS girls [21].

All blood samples were analyzed in the same laboratory. Serum DHEAS concentrations and follicle-stimulating hormone (FSH) levels were measured in the TS girls using an automated chemiluminescence immunoassay system (Immulite, DPC Biermann GmbH, Germany) and calculated in μmol/L (ng/mL × 0.0027 = μmol/L). Our own data on DHEAS levels from 178 healthy female Caucasian children aged 3–17 years, measured with the same method, were used as control group [22].

### Statistics

The statistics software SPSS, Version 21 by IBM was used for data analysis. We used GraphPad Prism™ software version 7.00 for presentation. All tests were two-tailed tests; the level of significance was defined as *p* ≤ 0.05. In descriptive statistics, the Mean, Standard Deviation (SD), Median, Minimum and Maximum were calculated. We used the Kruskal-Wallis test together with Dunn’s Multiple Comparison Test to compare the DHEAS levels between both groups of girls with TS and the girls in the control group. The Mann-Whitney U test was used to compare the ages at which the different Tanner stages of pubic hair were reached in the girls with TS.

## Results

### DHEAS

**Fig. 1:**
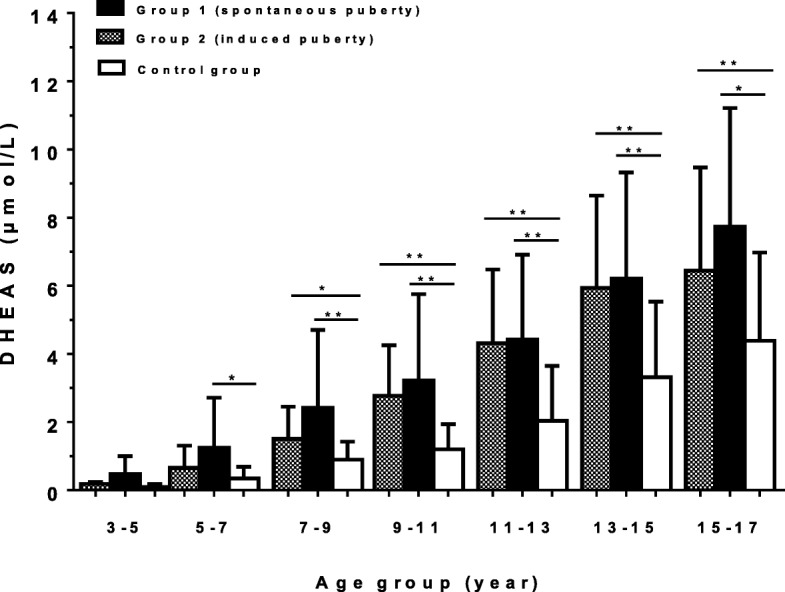
Serum DHEAS levels (mean ± SD) for age in Turner syndrome girls (black bars: group 1 with spontaneous puberty; hatched bars: group 2 with induced puberty; open bars: control group). The values are shown in μmol/L. For conversion to conventional units: μmol/L: 0,027 = μg/dL. Level of significance is indicated by asterisks (* * *p* < 0.01; * *p* < 0.05)

DHEAS levels are shown in Fig. 1. The DHEAS values (mean ± SD) in group 1 (spontaneous puberty) were 1.2 ± 1.5 μmol/L in the age group 5–7 years, 2.4 ± 2.3 μmol/L in the age group 7–9 years, 3.2 ± 2.5 μmol/L in the age group 9–11 years, 4.4 ± 2.5 μmol/L in the age group 11–13 years, 6.2 ± 3.1 μmol/L in the age group 13–15 years and 7.7 ± 3.5 μmol/L in the age group 15–17 years. Levels in group 2 (primary ovarian insufficiency, POI) were 1.5 ± 0.9 μmol/L in the age group 7–9 years, 2.8 ± 1.5 μmol/L in the age group 9–11 years, 4.3 ± 2.2 μmol/L in the age group 11–13 years, 5.9 ± 2.7 μmol/L in the age group 13–15 years and 6.4 ± 3.0 μmol/L in the age group 15–17 years. DHEAS levels in the girls in group 1 were slightly higher than those in group 2. DHEAS levels were elevated in all TS girls aged ≥5 years compared with the reference cohort. The TS girls in both groups showed significantly higher DHEAS levels in the age group 7–17 years than in the reference cohort. DHEAS levels in the TS girls in group 1 (spontaneous puberty) were significantly higher in the age groups 5–7 years (*p* < 0.05), 7–9 years, 9–11 years, 11–13 years, 13–15 years (*p* < 0.01), and age group 15–17 years (*p* < 0.05) than in the reference cohort. Significant differences between the TS girls in group 2 (induced puberty) and the control group were found in the age groups 7–9 years (*p* < 0.05), 9–11 years, 11–13 years, 13–15 years, and 15–17 years (*p* < 0.01).

### FSH

At Tanner B2, the serum FSH levels (mean ± SD) of the TS girls in group 1 were 6.34 ± 3.12 IU/L, whereas the TS girls without spontaneous puberty had significantly elevated FSH levels in the age group 11–13 years with 85.8 ± 35.4 IU/L.

### Puberty

Spontaneous puberty (Tanner B2) started in the TS girls in group 1 on average at 11.9 ± 1.2 (SD) years of age. Puberty was induced with estrogens in the girls in group 2 (POI) at the age of 13.7 ± 1.3 (mean ± SD) years, and Tanner B2 was reached significantly later (*p* < 0.001) at 14.3 ± 1.5 years. Menarche occurred earlier in group 1 at 13.7 ± 1.3 years of age than in group 2 at 15.5 ± 1.5 years (*p* < 0.001).

The age of the TS girls on reaching Tanner PH 2 to PH 5 is shown in Table 1. Pubarche occurred on average at the age of 10.5 years in the group of TS girls with spontaneous puberty and at 11.3 years in the group of TS girls with POI. The TS girls in group 1 with spontaneous puberty reached the Tanner stages PH2 (*p* < 0.04), PH3 (*p* < 0.01), PH4 and PH5 (*p* < 0.001) markedly earlier than the TS girls in group 2. In normal-weight German girls, the mean age was 10.2 years at PH2, 11.7 years at PH3, and 12.2 years at PH4 and 13.5 years at PH5 [21]. Only the data of the TS girls in group 1 conformed to the data of the KIGGS study [21].

**Table 1:** Chronological ages (years) of girls with Turner syndrome at the different Tanner stages of pubic hair PH 2–5; values given as mean ± SD, median (range)

Tanner stage	Group 1	Group 2	Level of significance group 1 vs. group 2
PH 2	10.5 ± 1.01 10.5 (9.0–12.0)	11.3 ± 1.62 11.0 (8.2–16.0)	*P* < 0.04
PH 3	11.8 ± 1.04 12.0 (10.4–13.4)	12.9 ± 1.96 12.6 (9.5–21.9)	*P* < 0.01
PH 4	12.4 ± 0.91 12.7 (10.9–13.6)	14.3 ± 1.82 13.9 (10.7–22.2)	*P* < 0.001
PH 5	13.8 ± 1.05 13.8 (11.9–15.4)	15.5 ± 1.61 15.2 (13.1–23.5)	*P* < 0.001

Group 1: spontaneous puberty; Group 2: induced puberty (primary ovarian insufficiency)

## Discussion

It has been shown that approximately 80% of girls with Turner syndrome (TS) show no spontaneous pubertal development, due to gonadal dysgenesis associated with gonadal failure [5]. The prevalence of spontaneous puberty varied in TS girls with karyotype 45 X between 6% [23] and 31% [4]. TS girls with primary ovarian failure usually have markedly elevated serum FSH levels [24–26] together with low serum anti-Mullerian hormone concentrations [27].

Since gonadarche and adrenarche are regarded as processes that are independent of each other [13–15], it has been assumed that adrenarche might not be affected in girls with TS and primary ovarian insufficiency (POI). In the literature data on adrenarche are scarce and inconsistent. For instance, 24-h urine samples showed elevated DHEA levels in 22 girls with TS compared with healthy girls [16], while Sklar et al. found normal serum DHEAS levels in 39 girls with TS and POI [15]. According to a study from Tübingen, adrenarche occurs markedly earlier in TS girls with POI (*n* = 45; median age: 8.3 yrs.) than in girls with spontaneous puberty (*n* = 22); median age: 10.5 yrs.) [18]. However, the study does not state whether the girls received growth hormone (GH) therapy.

It has also been speculated that treatment with growth hormone (GH) might affect adrenarche. Analyses of adrenal cell cultures have shown that both GH and IGF1 cause an increase in adrenal steroid biosynthesis [28] and IGF1 affects cell proliferation and reticular zone migration [29]. In the present study, all TS girls were treated with GH; we did not include any TS girls who did not undergo GH treatment.

The type of karyotype did not influence the time of adrenarche/pubarche. Both in our study and in the Tübingen cohort, adrenarche occurred in the TS girls in the age group of 5–7 years. At this age, the TS girls with spontaneous puberty and POI in our cohort already showed higher DHEAS levels than the reference cohort. However, significant differences between the TS girls in both groups and in the reference cohort only emerged in the age group 7–17 years. We did not find the significant differences in DHEAS levels between the TS girls with spontaneous puberty and POI in the age group of 9–15 years which were reported by Martin et al. [18].

In the literature the age at pubarche (PH2) varies in healthy girls between 10.4 ± 1.2 years and 11.8 ± 0.98 years [30, 31]. In our cohort, pubarche occurred on average at the age of 10.5 years in the TS group with spontaneous puberty, and significantly later at 11.3 years in the TS group with POI. The tempo of development of pubic hair in TS girls with spontaneous puberty did not differ from that in the reference cohort [21], whereas the tempo of pubarche was different in TS girls with POI who reached the relevant Tanner stages of pubic hair (PH3 - PH5) markedly later. Our data on pubarche confirm the results from Tübingen, which report statistically significant late pubarche at a mean age of 13.0 years in TS girls with POI compared with 11.9 years in the group with spontaneous puberty [18]. The authors suggest that normal pubarche is the clinical manifestation of the ovarian conversion of DHEAS to active androgens. We could confirm the results on pubarche, but we did not find any differences in the DHEAS levels measured.

The start of estrogen treatment (median age) in TS girls with POI was similar in Erlangen (13.7 years) and Tübingen (13.9 years), thus we exclude an estrogen effect on pubarche. Some of the differences could be explained by the reference cohorts used. However, the delayed development of Tanner stages of pubic hair in TS girls with POI suggests an ovarian effect on pubarche, as speculated in the study by Martin et al. [18].

## Conclusions

Some limitations of the present study should be considered. First of all, we have no data on a group of TS girls without GH treatment. Secondly, the sample size of the TS girls with spontaneous puberty is relatively small. Our data show that the onset of adrenarche in girls with TS undergoing GH therapy does not differ from that in healthy girls. However, adrenarche is exaggerated in girls with TS. There are no differences in DHEAS levels between the TS girls with spontaneous puberty and the TS girls with primary ovarian insufficiency (POI), while the tempo of pubarche is markedly slower in the girls with POI.

## Abbreviations

BMI: Body Mass Index
DHEA: Dehydroepiandrosterone
DHEAS: Dehydroepiandrosterone sulfate
POI: Primary Ovarian Insufficiency
TS: Turner Syndrome
